# Strong bending of the DNA double helix

**DOI:** 10.1093/nar/gkt396

**Published:** 2013-05-15

**Authors:** Alexander Vologodskii, Maxim D. Frank-Kamenetskii

**Affiliations:** ^1^Department of Chemistry, New York University, New York, NY 10003, USA and ^2^Department of Biomedical Engineering, Boston University, Boston, MA 02215, USA

## Abstract

During the past decade, the issue of strong bending of the double helix has attracted a lot of attention. Here, we overview the major experimental and theoretical developments in the field sorting out reliably established facts from speculations and unsubstantiated claims. Theoretical analysis shows that sharp bends or kinks have to facilitate strong bending of the double helix. It remains to be determined what is the critical curvature of DNA that prompts the appearance of the kinks. Different experimental and computational approaches to the problem are analyzed. We conclude that there is no reliable evidence that any anomalous behavior of the double helix happens when DNA fragments in the range of 100 bp are circularized without torsional stress. The anomaly starts at the fragment length of about 70 bp when sharp bends or kinks emerge in essentially every molecule. Experimental data and theoretical analysis suggest that kinks may represent openings of isolated base pairs, which had been experimentally detected in linear DNA molecules. The calculation suggests that although the probability of these openings in unstressed DNA is close to 10^−5^, it increases sharply in small DNA circles reaching 1 open bp per circle of 70 bp.

## INTRODUCTION

Although DNA double helix is pretty rigid, its bending is crucially important for the molecule functioning. DNA experiences bending either owing to thermal fluctuations or under the influence of special proteins, which bend DNA upon the binding or when DNA is forced to form a circle. In this review, we deal only with naked DNA, without any protein participation. Bending of naked linear and circular DNA molecules has long been described by the wormlike chain (WLC) model, which have become a major theoretical tool making it possible to quantitatively describe a huge body of various experimental data, such as topological properties of circular DNA, single-molecule experiments on DNA stretching, DNA cyclization, and many more [reviewed in (1–3)]. In 2004, the conventional view on DNA bending was challenged by Cloutier and Widom (C&W) who claimed that cyclization of short DNA fragments, around 100 bp long, occurred about 1000 times more efficiently than the WLC model predicted (4). Since then, bending of short DNA fragments has attracted a lot of attention (5–24). The goal of this review consists in sorting out what is real and what is not about strong bending of the double helix. We show that there is nothing mysterious in this phenomenon—strong bending of DNA has to be facilitated by kinks of the double helix. This property of DNA bending has been predicted many years ago by Crick and Klug (25). It is important to emphasize that appearance of such kinks in strongly bent DNA segments is not a property of short DNA fragments; it is equally possible in large DNA molecules. An important quantitative question here is the characteristic curvature of the double helix that causes formation of kinks. We analyze here experimental and theoretical approaches to this question and the answers that have been obtained. As a result of this analysis, we arrive in this article at a coherent picture of the mechanism of strong DNA bending, which explains all reliable data on the issue.

The most natural way to strongly bend DNA is converting it into a circular form. The efficiency of cyclization process is quantitatively described by the so-called *j*-factor [due to Jacobson and Stockmayer who pioneered in considering the polymer cyclization phenomenon (26)], the effective equilibrium concentration of one end of the DNA fragment in the vicinity of the other end. In case of double-stranded DNA, the *j*-factor usually also accounts for torsional and axial alignment of the juxtaposed ends of the molecule. It is critical for our understanding of DNA bending that the *j*-factor can be calculated for various bending models and can be measured experimentally. The concept of the *j*-factor will be widely used throughout this review.

## MODELING OF DNA BENDING

It has been commonly accepted over the past few decades that DNA bending occurs by small changes of the angles between the planes of adjacent base pairs and can be quantitatively described by the WLC model. In this model, which was originally proposed to describe semi-flexible polymeric molecules by Bresler & Frenkel (27) [see also (28)], the bending energy of the chain, 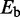, is calculated as
(1)
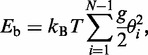

where *k*_B_ is the Boltzmann constant and *T* is the absolute temperature, *g* is the dimensionless bending rigidity constant that is defined by DNA internal properties, 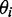 are the angles between the directions of segments *i* and *i* + 1, *N* is the number of segments in the molecule. The *g* constant is proportional to the chain persistence length, *a*:
(2)
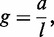

where *l* is the length of the chain segments (the distance between adjacent base pairs in case of DNA). It is worth to consider the assumptions and approximations made by modeling DNA conformations via WLC.

**Figure 1.**
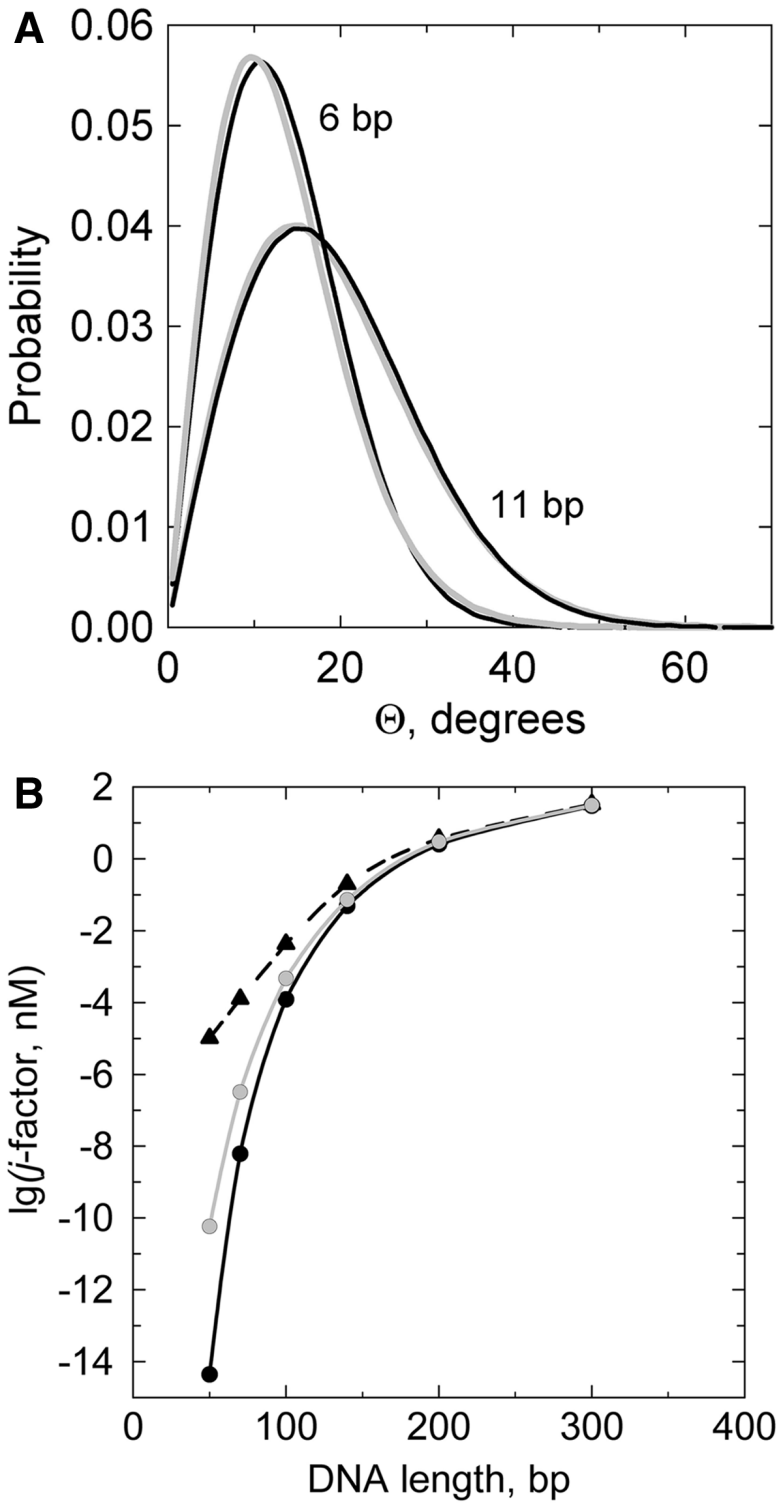
The effect of the bending potential on DNA conformational properties. (**A**) The distributions function 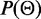 for angle Θ between two unit vectors tangential to the two DNA termini for chains with quadratic (black lines) and linear (gray lines) bending potentials. (**B**) The *j*-factors for the chains with quadratic (black line) and linear (gray line) potentials and for quadratic potential with kinks (dashed line). The bending potential that allows for kink formation was modeled by Equation (3) with *h* of 12. The shown *j*-factors do not allow for the torsional orientation of the fragment ends. The bending rigidity constants for all potentials were chosen so that the *a* value was the same for all models (48 nm). The computer simulations are described in detail in (1).

(i) Within the WLC model, the minimum energy of the chain corresponds to zero bend angles between adjacent base pairs. In reality DNA can have some intrinsic curvature, so the minimum energy conformation of the DNA axis may deviate from the straight line. Of course, the intrinsic curvature has to be specified by the DNA sequence. However, with the exception of special sequences, the contribution of intrinsic bends into the *a* value is small (29). An essential intrinsic curvature is mainly associated with A-tracts [reviewed in (30–32)]. We will not consider this issue here.

(ii) The flexibility of WLC is isotropic, that is the bending energy is independent of the bend direction. Obviously, it is not the case for the double helix for which the bending rigidity in the directions to the helix grooves, 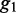, is a few times smaller than the bending rigidity in the perpendicular directions, 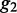 (33). In addition to this, there is no reason to believe that the bending rigidities in the directions of the major and minor groves of the double helix are the same. A model allowing for the bending anisotropy was suggested in (34). However, if we are interested in conformational properties of DNA segments larger than 20–40 bp in length, there is no need to allow for this anisotropy. The helical structure of DNA causes the fast self-averaging of the bending anisotropy, so it is not essential for large-scale properties of DNA molecules (35,36).

(iii) The bending rigidity of WLC does not change as one moves along the chain. In terms of DNA, it means that the bending rigidity of the double helix does not depend on the DNA sequence. Definitely, this is an approximation; DNA bending rigidity does depend on its sequence, although the effect is relatively small. It was determined that the rigidity for individual base pair steps deviates from the average value by no more than 20% (37). In a DNA fragment with a typical sequence, the self-averaging of *g* occurs in the scale of a few helix turns.

(iv) The quadratic bending potential as a function of θ is usually assumed in the WLC model [Equation (1)]. This assumption deserves more attention here. The quadratic bending potential corresponds to the first non-zero term of the Maclaurin expansion of the exact potential, if one assumes that the bending energy has a minimum at θ = 0. It is worth to note, however, that the choice of the potential is not important for the bending properties of DNA in the scale of a few helix turns. Indeed, due to the Central Limit Theorem of the probability theory, the probability distribution 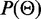 for angle Θ between two unit vectors tangential to the two termini of a DNA fragment larger than 10 bp in length is well approximated by the Gaussian distribution function for nearly any bending potential. This is illustrated in Figure 1A, which shows the 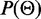 distribution calculated for two model chains with quadratic and linear bending potentials. The distribution 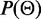 corresponds to the spherical coordinate system where the number of states of the fragment that corresponds to angle Θ increases with the angle increase as sinΘ [see (38), for example]. This is why the distribution maximum is at nonzero magnitudes of Θ. The bending rigidities of the model chains in Figure 1A are chosen so that they had the same *a* value. We see from the figure that the 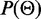 function is slightly different for two models for the chains consisting of 6 bp, but this difference nearly disappears for the chains consisting of 11 bp. Although such variations of the potential are more important for strongly bent conformations, even in these cases the variations in the calculated properties remain relatively small. This is illustrated in Figure 1B where the *j*-factors for the two model chains are shown. Clearly, even for model chains that correspond to 70 bp in length, the values of the predicted *j*-factors are relatively close. We have to make an important conclusion that modification of the bending potential for small bend angles cannot result in a significant increase of the *j*-factors for short DNA fragments.

Although a larger difference in the calculated properties of short fragments was obtained for the model with linear bending potential suggested in (8), it had an additional assumption. The authors suggested that the straight segments of the model chain consist of 7 bp. Clearly, the latter assumption reduces the effect of the Central Limit Theorem on the short fragments. It is difficult, however, to justify either of the two assumptions of such model.

It was suggested by Crick and Klug that sharp kinks of the double helix, which maintain base pairing but disrupt the stacking interaction between two adjacent base pairs, can be energetically favorable way to make strong bends of the double helix (25). Although such kinks have to be energetically costly, they can essentially reduce the bends and, correspondingly, the bending energy in the neighboring stacks of base pairs. The probability of kink formation in the absence of bending stress should be low, so it does not notably affect the angle Θ distribution function (see Figure 1A). Crick and Klug estimated, by using a simple wire model, that the bend angle in such kinks can slightly exceed 90° (25), and the estimation was later confirmed by molecular dynamic simulation (9). Examples of kinks of 100° and greater can be found in the database of DNA–protein complexes (39). Similar, but not identical, to Crick & Klug kinks are kinks associated with base pair openings. Probably, energetic cost of such disruptions should be even higher than for Crick & Klug kinks. However, the disruptions that include open base pairs should provide even more local flexibility and could be a preferable way of changing twist of the double helix, if a stressed conformation requires it.

**Figure 2.**
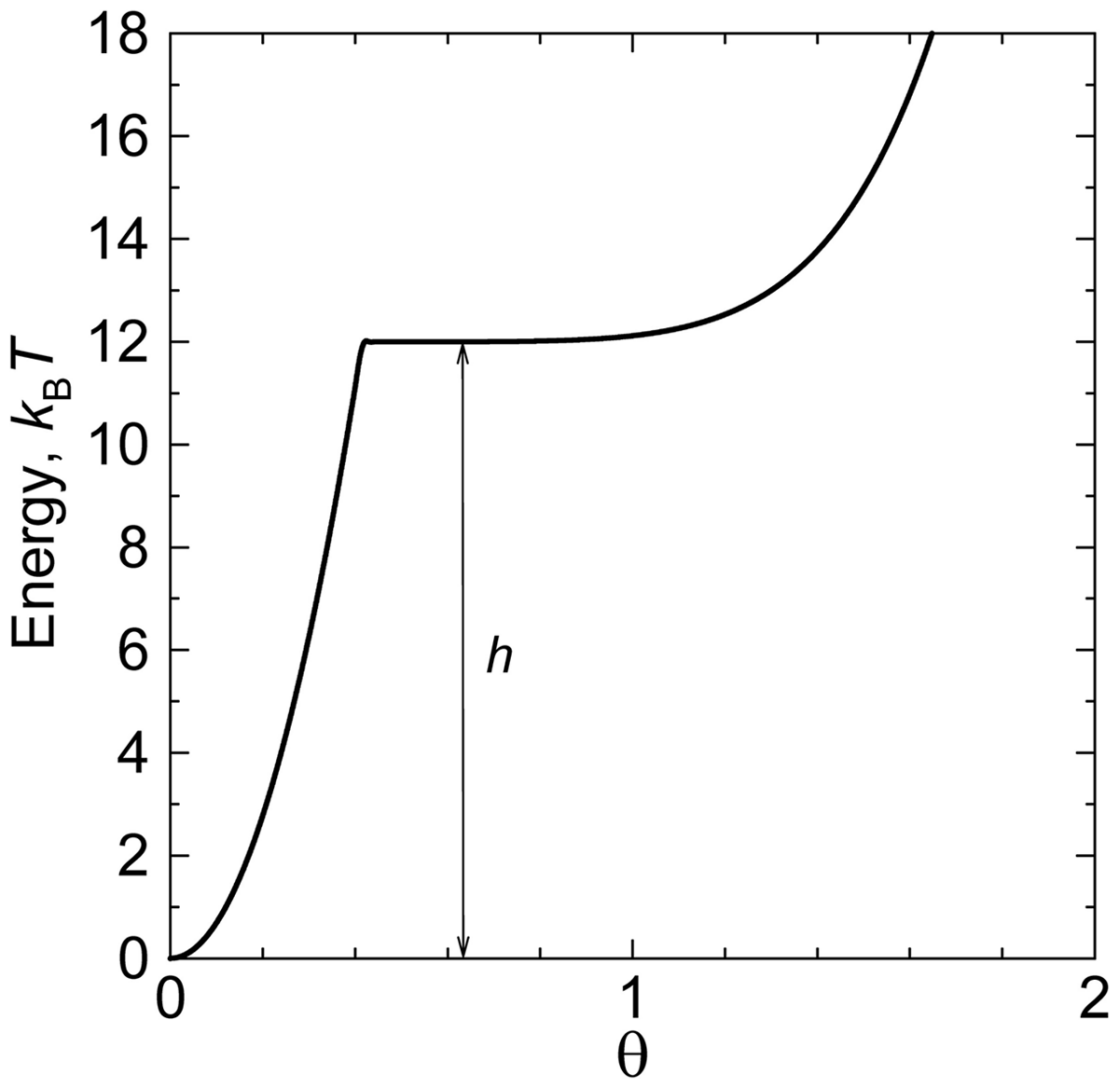
The bending potential that allows for a possibility of kinks with a bend angle up to 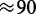
° (Equation 3). The value of *b* specifies the width of the plateau that corresponds to kinks (*b* = 0.3 in the shown case); the *h* value (12 in the shown curve) specifies the energy of the kink formation. The free energy of the kink is ∼5*k*_B_*T* lower than the magnitude of its energy, *hk*_B_*T* (see text).

The theoretical analysis clearly shows that kinks are capable of increasing the *j*-factors of short DNA fragments by a few orders of magnitude (5–7). If we knew the energy associated with the kink formation as a function of the bend angle, we could unambiguously estimate at what curvature of the double helix the kinks should appear. Since the function is not known, we will assume that energy profile at the kink is flat till certain value of the bend angle. A simple functional form for such bending potential will be used here:
(3)
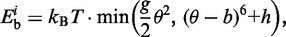

where *h* is a parameter that defines the energetic cost of the kinks, and *b* specifies the range of allowed bend angles. With *b* = 0.3, this potential allows for bend angles at the kink up to ∼90° (Figure 2). It should be noted that owing to a high flexibility of DNA at kinks, a much larger array of bend angles is accessible at the kink than at undisrupted stacks of base pairs [see above remark regarding 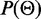]. Therefore, the free energy of kink formation is substantially lower than its energy *hk*_B_*T* (40). Of course, this effect is automatically accounted for in the simulation of equilibrium conformational properties (1).

The computed values of the *j*-factors for *h* = 12 are shown in Figure 1B (this *h* value was chosen for the illustration purposes only). One can see from the plot that for DNA fragments of 70 bp in length, the *j*-factor increases by >4 orders for this value of *h*. Thus, we conclude that kinks facilitate strong bends in the double helix and, in particular, greatly increase the *j*-factors of very short DNA fragments. The only remaining question is what is the characteristic DNA curvature where the contribution of kinks becomes important.

**Figure 3.**
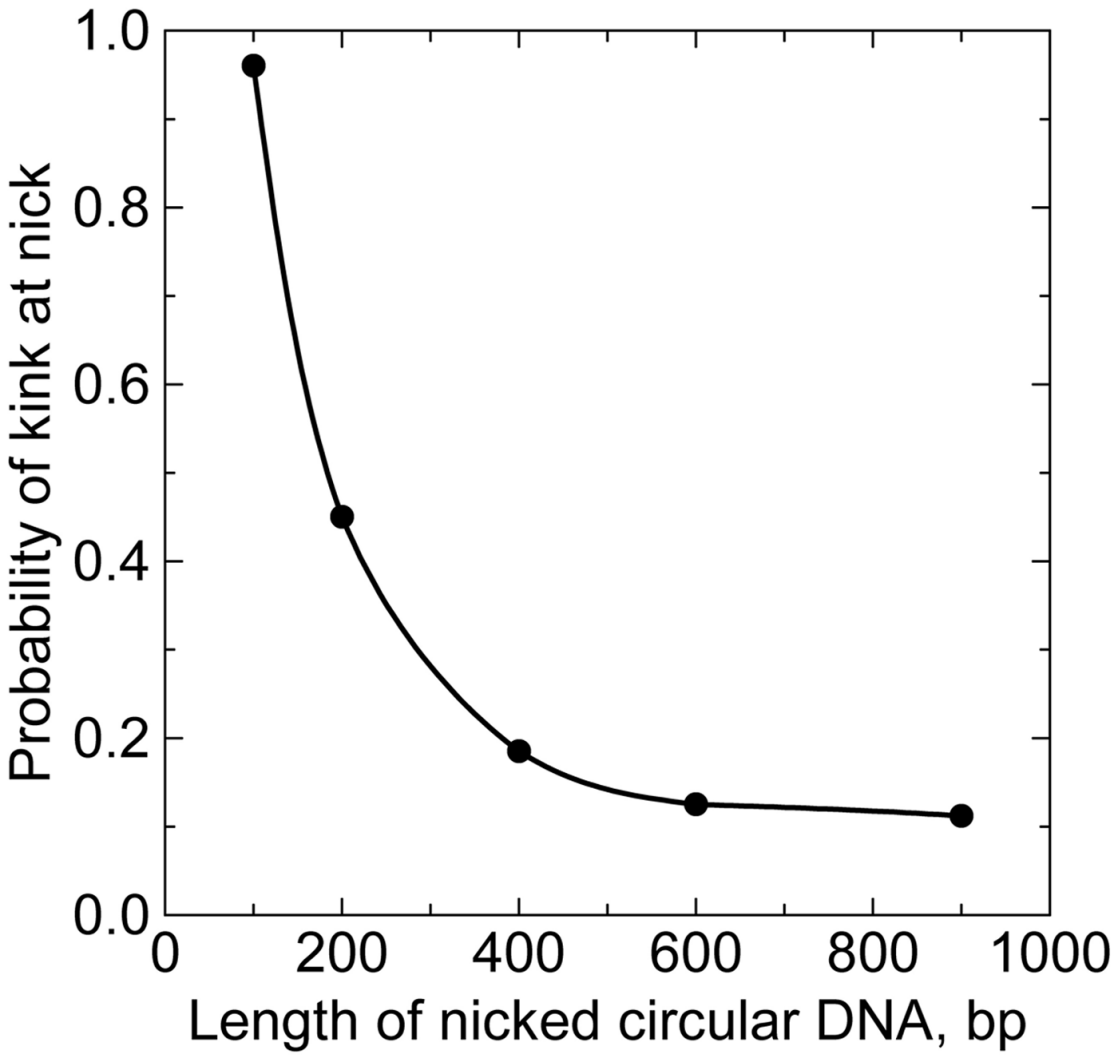
The probability of the kink appearance at the nick in circular DNA. The data obtained by computer simulation of equilibrium set of DNA conformations with the bending potential at the nick described in the text.

The energetics of the kink formation has been studied in detail at the sites of single-stranded DNA breaks (nicks) (41,42). These studies clearly showed that at nicks the free energy of the kink formation is low. Because the kink formation at nicks is important for the problems in this review, we used the available data to investigate the issue by computer simulation. We constructed the bending potential at nick similar to one described by Equation (3), although allowing the bends up to 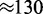
° at nicks (*b* = 1.0). With this value of *b*, the value of *h* was chosen so that the probability of kinks matched the experimental observations (41,42). By this way, we found that the proper value of *h* equals to 8. This value was obtained by averaging over different base pair steps and corresponds to 0.2 M of [NaCl] in accordance with (41,42). Using the computer simulation of circular DNA molecules with a nick, we found the probability of the kink at the nick as a function of the circle size (Figure 3). The figure shows that in nicked DNA circles of ≤100 bp, the kink is always formed at the nick. In particular, the kink permanently exists in DNA circles of these sizes that were formed by joining sticky ends of a linear fragment. This conclusion has to be taken into account when we analyze cyclization of short DNA fragments. One can also see from the figure that the probability of the kink formation approaches ∼0.1 as the circle size exceeds 600 bp, in agreement with the data for linear DNA fragments (41,42). It should be noted that this probability is in qualitative agreement with earlier studies of DNA conformation at nicks (43,44).

## EXPERIMENTAL AND COMPUTATIONAL APPROACHES

### *j*-Factors of short DNA fragments assessed by ligation

DNA cyclization by ligation as a method of the *j*-factor determination was introduced by Baldwin and co-workers in 1981 (45). By this method, Shore and Baldwin obtained an elegant estimation of the DNA helical repeat in solution, when they observed the oscillation of the *j*-factor of short DNA fragments with the change of their length (46). They performed a careful analysis of the approach and showed that the method allows the *j*-factor determination only if certain conditions are met. It is important to consider these conditions here.

**Figure 4.**
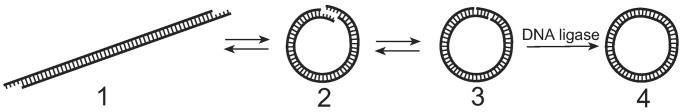
Successive states of the DNA fragment during its conversion from linear to closed circular form with the aid of the DNA ligase. In state one, the sticky ends are apart one from the another; in state two, the sticky ends are juxtaposed but not joined; in state three, they are joined by base pairing; in state four, the joined sticky ends are ligated.

The process of cyclization involves a few steps diagrammed in Figure. 4. The first step is juxtaposition of the fragment ends with proper mutual orientation (state 2). The probability of this state is proportional to the *j*-factor that has to be determined in the assay. The juxtaposed sticky ends can be joined by forming base pairs (state 3). It was shown by Wang and Davidson that the rate of the sticky ends joining is not limited by the rate of their first collision (47), so that many collisions of the sticky ends precede their joining. Therefore, the rate of the sticky ends joining is proportional to the equilibrium fraction, 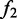, of the fragment conformations with juxta-posed sticky ends (state 2). Of course, this is a necessary condition to measure the *j*-factor by measuring the cyclization rate. It seems that the condition is always satisfied. However, the rate of the sticky ends joining is not determined in the assay. Instead, the assay can only determine the equilibrium fraction of the molecules with joined sticky ends, 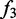 (state 3). If 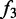 is small and corresponds to the thermodynamic equilibrium, it is also proportional to 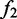. This is possible only if the rate of the sticky ends ligation is much lower than the rate of the sticky ends dissociation (transition from state 3 to state 2), so that many joining–dissociation events precede the ligation. This condition is satisfied only if the ligase concentration is sufficiently low and the sticky ends are sufficiently short. The latter condition is also necessary to keep 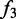 small. If these conditions are met, the rate of the fragment conversion into closed circular form, catalyzed by DNA ligase, is proportional to its *j*-factor. The proportionality coefficient is determined in the method by comparison with the rate of the fragment dimerization in the same experiment:
(4)
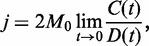

where 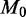 is the initial concentration of the fragments in solution, *C*(*t*) and *D*(*t*) are the concentration of ligated monomeric circles and dimers, respectively, at time moment *t* [see (3,29,48) for details]. The normalization of the cyclization rate by the rate of dimerization carries an additional assumption: the conformational distribution of DNA segment with joined unligated sticky ends has to be the same in the circles and in the dimers.

**Figure 5.**
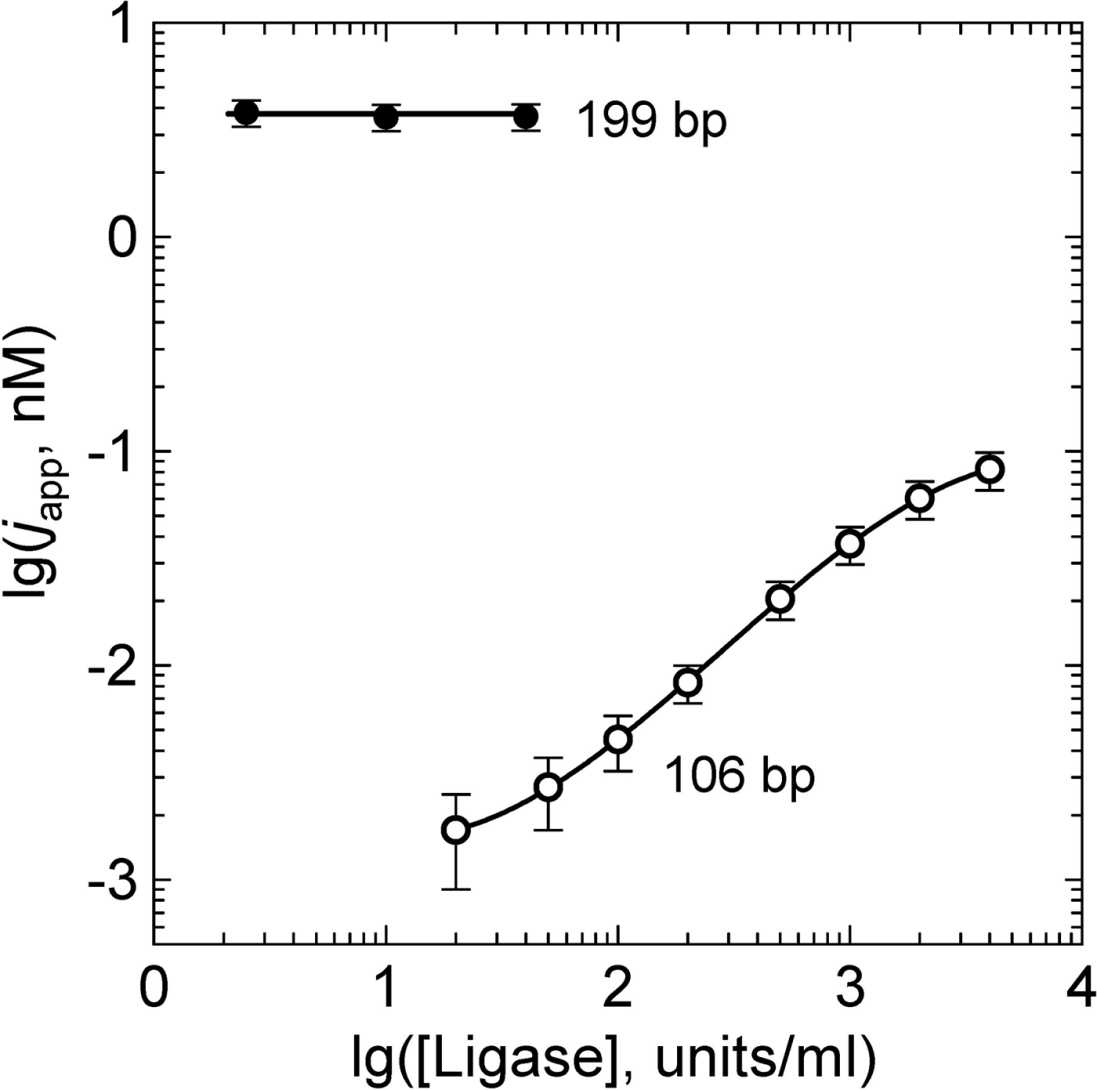
Dependence of the apparent *j*-factor, *j*_app_, on the ligase concentration, [ligase]. The method requires that [ligase] would be low enough, so that the measured *j*_app_ does not depend on [ligase]. The shown data correspond to the permissive range of [ligase] for 199-bp fragment but not for 106-bp fragment (7). The [ligase] is given in the units of New England Biolabs.

The ligase-assisted DNA cyclization allows a remarkably accurate determination of the fragment persistence length by comparing the experimental data with the theoretical and computer calculations of the *j*-factor [see (37), for example]. However, problems emerge when the method is applied to very short DNA fragments that have very small *j*-factors. In such cases, the accumulation of the ligated circles occurs at the time scale comparable with the time of ligase activity (about 2 h at room temperature). This prompts to use higher ligase concentrations that may violate the assay conditions (Figure 5). It is exactly what happened in case of the C&W study that initiated the current interest in the issue of strong DNA bending (4). In addition to high ligase concentration, C&W used quite stable sticky ends that form four GC pairs. Even at 30°C, the dissociation time of these sticky ends is in the scale of minutes (49). C&W concluded that the *j*-factor of DNA fragments 96–126 bp in length was about three orders of magnitude higher than the values predicted by the WLC model. Du *et al.* (7) found, however, that the overestimated values of the *j*-factor obtained by C&W were due to very high ligase concentration they used in their study. Under permissive concentrations of DNA ligase, the *j*-factors of DNA fragments of 105 bp and larger were in excellent agreement with the prediction of the WLC model (7).

There is another problem with the ligase-assisted cyclization of very short fragments, however. The probability of kinks at nicks surrounding the joined sticky ends is relatively high even in the absence of the bending stress. When the bending stress in small circles increases, the probability of kinks at nicks rises fast (see Figure 3). Thus, conformations of joined sticky ends in the circles and linear dimers become increasingly different, and Equation (4) cannot be used, strictly speaking, for the *j*-factor determination. The kinks should affect the dissociation rate of the joined sticky ends, and, may affect the ligase activity. Although it is difficult to estimate the effect of these factors quantitatively, it seems that the overall affect is negligible for the fragments of 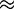
200 bp in length or longer, but it should become notable for fragments of 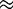
100 bp. Thus, the ligase-assisted cyclization of DNA fragments with the length around and below 100 bp is not the best way to address the problem of the critical DNA curvature that causes formation of kinks.

### Cyclization of DNA fragments with long sticky ends

**Figure 6.**
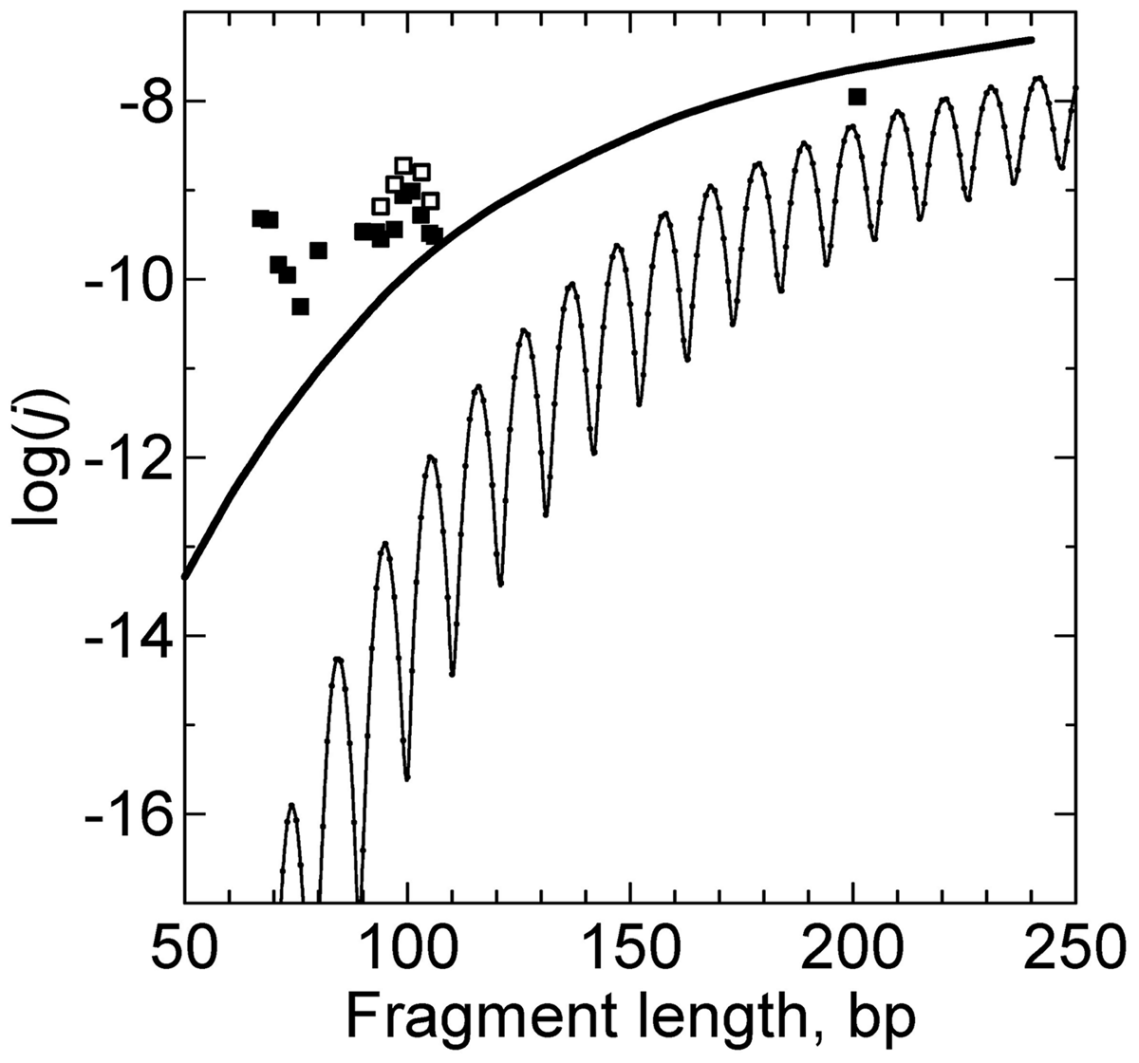
The *j*-factor for fragments with long sticky ends. Long sticky ends, ≥8 nucleotides, can form a stable double-stranded segment at any mutual axial and torsional orientations of the double-stranded ends. Also, the separation between the double-stranded ends, *r*_0_, during the joining can be notable. The solid curve corresponds to the theoretical calculations based on the WLC model and *r*_0_ of 5 nm. The thin line corresponds to the *j*-factors for the WLC model with requirements of axial and torsional orientations (short sticky ends). The symbols are experimental data from Figure 2 of (23), obtained for surface-tethered DNA (filled squares) and vesicle-encapsulated DNA molecules (open squares).

The first experimental determination of the *j*-factors of DNA molecules was performed by Wang and Davidson who used phage DNA molecules with long natural sticky ends (50). They showed, in particular, that the rate of joining of the sticky ends is proportional to the equilibrium concentration of one sticky end in the vicinity of the other. Therefore, the *j*-factors can be determined from the ratio of the rates of the circles and dimers accumulation (50). At low temperature, both association and dissociation of long sticky ends (8 and more nucleotides) proceed very slow (47), so it is not a problem to measure the kinetics of accumulation of the circles and dimers. Because the method does not require ligation of the joined ends, it represents a more straightforward way of the *j*-factor determination. Its application to the cyclization of very short DNA fragments has one difficulty, however. Owing to very small value of the *j*-factor of the fragments, their molar concentration has to be very low, so that the fractions of formed circles and dimers will be comparable. It is not a problem if one works with DNA molecules of many thousands base pairs in length, as they still can be easily detected. If the fragments are short, however, it is difficult to measure their amount at low molar concentration. To overcome this difficulty, Vafabakhsh and Ha (23) attached fragments, end-labeled with donor and acceptor, to a grid under conditions where they could not form dimers and observed the cyclization in the individual molecules by FRET. The approach requires chemically modified DNA fragments and therefore better fits to synthetic rather than cloned or PCR-produced DNA fragments. It has been argued, however, that synthetic fragments are not suitable for this kind of experiments, as they have insufficient purity [(51); see also (52) and Supporting Material of (12)]. Sequence errors in synthetic oligos result in mismatches and bulge loops in the duplexes that serve as hinges, facilitating the cyclization. It is also important to keep in mind that joining long sticky ends does not require axial and torsional alignment of the DNA ends, as long single-stranded sticky ends are very flexible and can form a double-stranded segment at any mutual orientation of the duplex ends. For DNA fragments of ≤120 bp, the *j*-factors for arbitrary orientation of the ends are very different from the *j*-factors that account for perfect axial and torsional alignment (Figure 6). These factors suggest that the claim made by Vafabakhsh and Ha about ‘extreme bendability of DNA less than 100 base pairs long’ is hardly supported by their experimental data (51). Therefore, so far the method has not produced a reliable estimation of the critical DNA curvature for the kink formation, although it looks promising in this respect.

### Nuclease probing of covalently closed minicircles

Single-strand-specific nucleases have been widely used to detect disruptions and alternative structures in negatively supercoiled DNA molecules (53–57). These enzymes introduce single-stranded cuts (nicks) at DNA sites with structural deviations from the regular double helix. Du *et al.* (12) applied the approach to detect disruptions in covalently closed DNA minicircles 63–106 bp in length. They found that in the absence of torsional stress, the circles of ≥84 bp are resistant to the nuclease treatment while the circles of 63–64 bp are digested by BAL-31 nuclease. The result suggested that kinks were not present in the minicircles of 84–85 bp is length, and could only appear in the torsionally unstressed circles of ≤74 bp. This means that bending stress disrupts the regular helical structure when the radius of DNA curvature is <3.5 nm. It is interesting that the disruptions were detected by the treatment by BAL-31 nuclease, but not S1 nuclease (12). The latter enzyme was capable to digest only negatively supercoiled minicircles, where the disruptions had to be more extensive. It prompted the assumption that the Crick & Klug kinks rather than opened base pairs appear in the torsionally unstressed minicircles of 63–64 bp in length (12).

Of course, negative supercoiling strongly promotes the disruption formation, as was studied in detail in 1980s [see (58) for review]. The minicircles are much more sensitive to the supercoiling, however, because in supercoiled state they remain flat and therefore have no writhe, while in large circular DNA writhe absorbs about 75% of the supercoiling. Thus, in the minicircles, the linking number deficit can only cause a reduction of DNA twist (59–61). For example, if the increase of the circle length from 64 to 65 bp introduces the superhelix density, σ, of −0.017, it is equivalent to σ of −0.068 in a large circular DNA. The negative supercoiling of the latter magnitude causes structural disruptions in large DNA molecules. Thus, careful accounting for the effect of the torsional stress on the kink formation is absolutely necessary in the case of covalently closed minicircles.

It looks like the nuclease probing has produced the most reliable, so far, estimation of the DNA critical curvature causing the kink formation. Still, we do not know precisely what disruptions are probed by the BAL-31 nuclease. So, it is definitely desirable to apply other approaches to the problem.

### Electron microscopy

Cryo-electron microscopy was applied to DNA minicircles in an attempt to visualize kinks directly (14). The resolution of the method was not sufficient to reliably detect individual kinks, so the authors of the study applied statistical approach to compare structures of gapped, nicked, and covalently closed conformations of 94 bp minicircles. They concluded that there were no kinks in the 94-bp-long minicircles, in agreement with (7,12). The detected difference between conformations of gapped minicircles, where sharp bends are unavoidable, and intact minicircles was relatively small, however.

### Molecular dynamic simulation

In principle, molecular dynamic simulations would be a method of choice to address this kind of problems, and Lankas *et al.* (9) applied it to simulate conformations of the minicircles. They performed the MD simulations of a 94-bp-long circle while treating explicitly the solvent. The simulations revealed, in particular, a new type of kink, which involved three consecutive base pairs. The hydrogen bonding of the two flanking pairs was intact, while the central pair was broken, and its bases were stacked on the 5′ neighboring bases of the corresponding strands. The bend angle at the kink angle was close to 120°. A few kinks with intact base pairs were also observed. The simulations were unable, however, to reproduce the known structure of the double helix accurately: DNA helical repeat in the simulated structure was equal to 12 bp/turn rather than 10.5 bp/turn (9). Therefore, it is not clear whether in the present form the simulations yield quantitatively reliable predictions of such challenging DNA properties as transient formation of disruptions. More accurate potentials and extensive comparison between simulations and experimental data are needed before the MD method will produce reliable and quantitative predictions of disruptions of the DNA helical structure under the bending stress.

## CONCLUSIONS AND FUTURE DIRECTIONS

We have to conclude that despite all the efforts, there is only limited success in obtaining the critical value of DNA curvature that causes formation of kinks in the double helix. What can be stated confidently is that in 100-bp-long circular DNA molecules (or longer) without torsional stress, kinks are not formed. The most reliable estimation of the critical curvature comes from probing the structure of DNA minicircles by single-strand-specific endonucleases (12). These data showed that the critical radius of DNA curvature should be close to 3.5–4.0 nm, which corresponds to DNA minicircles of 63–74 bp. It has to be noted, however, that only one of two endonucleases used in the assay, BAL-31, detects disruptions of DNA structure caused by the bending stress. It cannot be excluded that even this enzyme is not capable to digest DNA at some disruptions that appear in DNA circles between 63–74 and 100 bp. Thus, the above estimation of the critical radius of curvature should be considered as a lower limit. Definitely, more data on the issue are needed.

**Figure 7.**
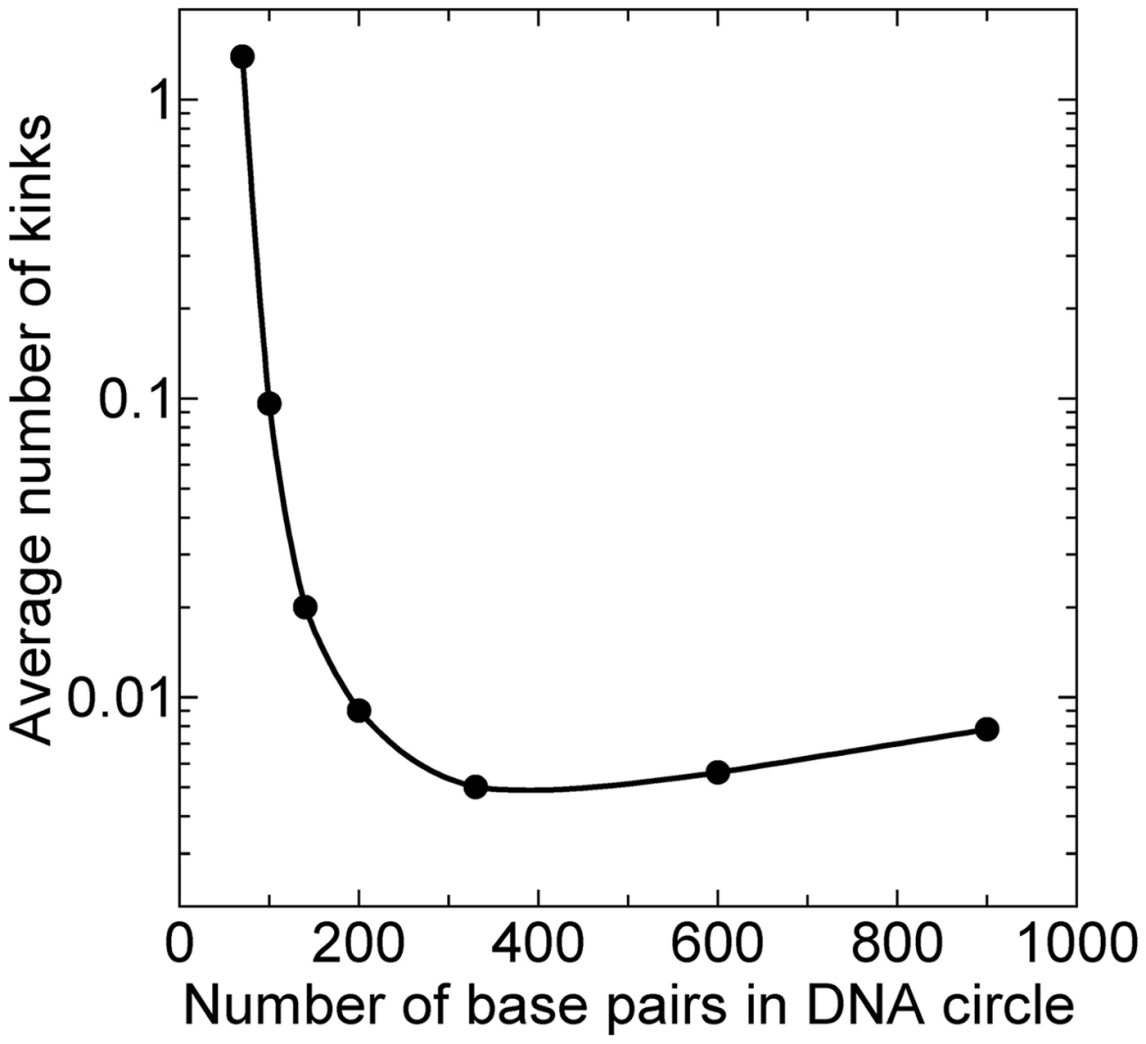
Computed average number of kinks (open base pairs) per molecule in circular DNA as a function of the number of bp in DNA. The bending potential at the disruptions correspond to Equation (3) with *b* = 0.3 and *h* = 16, which corresponds to the free energy of the disruption formation close to 7 kcal/mol, found experimentally (see the text). Changes in DNA torsional stress related to the disruption formation were not taken into account in the calculations that were performed as described in (1).

The probability of disruptions in DNA minicircles could be calculated in phenomenological models used for the *j*-factors estimation, and such analysis has been suggested (15). It cannot give a direct answer about the magnitude of the critical curvature, as the energetic and conformational parameters of DNA kinks are not known. We do not know the free energy of DNA kinks in unstressed DNA, and do not know how the kink energy depends on the bend angle at the kink. There are only limited data for base pair opening in linear DNA molecules [see (62) for review]. They are based on measurements of the proton exchange rate (63,64) and the rate of DNA unwinding by formaldehyde (65–67). According to these results, the free energy of a base pair opening is close to 7 kcal/mol for opening AT base pairs. If we assume that this free energy corresponds to formation of flexible hinges with the bending potential described by Equation (3), we find that the average number of kinks approaches 1 for DNA circles of 70 bp (Figure 7). Clearly, this is in a very good agreement with the data on the nuclease probing discussed above (12). Our data in Figure 7 make it very possible that the real nature of the double helix disruption, when short fragments are circularized, consists in the single base pair openings. However, there are no conformational data on DNA segments with opened base pairs detected by the cited methods. Thus, at present time we do not have sufficient information on structural and energetic properties of DNA disruptions to solve this challenging problem solely by the computations.

It was discovered recently that Hoogsteen base pairs appear in linear DNA with probability close to 0.01 (68). Although it looks like stacking with adjacent pairs is well preserved when Hoogsteen base pair is formed, it may still produce a point of higher flexibility that can facilitate strong bending of the double helix. Of course, this issue requires further studies.

## FUNDING

Funding for open access charge: Boston University.

*Conflict of interest statement*. None declared.
